# Influence of Pre-Strain on Static and Fatigue Properties of S420M Steel

**DOI:** 10.3390/ma16020590

**Published:** 2023-01-07

**Authors:** Stanisław Mroziński, Adam Lipski, Michał Piotrowski, Halina Egner

**Affiliations:** 1 Faculty of Mechanical Engineering, Bydgoszcz University of Science and Technology, Al. Prof. S. Kaliskiego 7, 85-796 Bydgoszcz, Poland; 2 Faculty of Mechanical Engineering, Cracow University of Technology, Al. Jana Pawła II 37, 31-864 Kraków, Poland

**Keywords:** pre-strain, low-cycle fatigue, durability

## Abstract

This paper reports the results of static tensile and low-cycle fatigue tests on S420M steel specimens. As-received (unstrained) and pre-strained specimens were used during the tests. Based on the static tensile tests carried out, no effect of pre-strain on the basic strength parameters of the S420M steel was found. Low-cycle fatigue tests showed that the pre-strain of the specimens causes a change in the cyclic properties of the steel and a slight increase in fatigue life compared to that of the as-received specimens. The greatest increase in durability was observed at the lowest strain levels.

## 1. Introduction

Structural components of many technical facilities, such as power plants, wind power stations, or steel bridge structures, may be subjected to static overloading in emergency situations as a result of seismic events, extreme weather conditions, etc. The risk of such events occurring is increasing, as WMO data shows that the number of natural disasters has increased fivefold in the last 50 years, causing losses of US$202 million per day [1]. The overloading can result in permanent plastic strain on the structure. The accumulation of strains from emergency situations, and strains resulting from alternating working loading, can cause premature failure of structural components and reduce their fatigue life. The effects of initial and accidental deformations are usually not taken into account in fatigue calculations, which are based on strength properties and characteristics determined using materials without initial deformations. Therefore, in order to effectively design many technical facilities, detailed knowledge of the material’s response to the additional strain occurring during the operation of the facility is essential.

Material properties are influenced by many factors related to loading, such as internal pressure, type of loading, environment, and temperature [2,3,4,5,6,7]. Of these, the pre-load (assembly load for example) is very important. Recently, extensive research into the straining of materials under pre-loading has been described in papers [8,9,10,11,12,13,14,15,16,17,18], among others. Yang and Wang [8] studied the effect of different pre-strains on the fracture of high-strength spring steel. The dependence of cyclic hardening or softening on the pre-strain was found. In the work of Taheri et al. [9], the detrimental effect of pre-strengthening achieved during pre-strain on fatigue life was found. Kim et al. [10], based on low-temperature testing of AISI 304L steel specimens, found that pre-strain results in increased yield strength and toughness at the expense of low-temperature ductility loss.

In [19], Mroziński and Szala, on the basis of low-cycle tests of C45 structural steel, showed that initial deformation causes a decrease in fatigue life and a new state of steel stabilization after deformation. The reduction in durability is affected by the size of the initial deformation set before the variable load. In [20] Brancoa et al. analyzed the influence of initial deformations on the fatigue life of the 7050-T6 aluminum alloy. The analysis was carried out using fatigue diagrams in terms of energy and deformation. Based on the analysis of the strain-life and energy-life diagrams, it was found that the fatigue life decreases with increasing initial strain. The reduction in durability is greater for higher levels of initial deformation.

The reduction of the fatigue life of the AA2024-T3 aluminum alloy due to initial deformation was also investigated by Wang in [21]. Contrary to the work in [20], a greater decrease in durability was found in the fatigue limit area. With the increase in the variable load, the reduction of fatigue life was much smaller.

In [22] Robertson et al. studied the fatigue properties of pre-deformed TRIP 780 multiphase steel samples. Contrary to the papers [20,21], it was found that the initial deformation followed by quenching resulted in an increase in the durability of the steel for variable deformations greater than 0.004.

In [23] Ly and Findley also analyzed the effect of pre-deformations of TRIP780 multiphase steel samples carried out at different temperatures (−20 °C, 0 °C, 80 °C). A small dependence of fatigue life on the pre-strain temperature was found.

Based on an analysis of the literature data, it can be concluded that pre-strains occurring prior to alternating loading most often result in a reduction in fatigue life [24,25,26,27]. They can also significantly alter the cyclic properties of the material. The effect of pre-strain on the reduction in durability and the change in cyclic properties of the material depends on both the amount of pre-strain and the level of alternating load occurring afterward. In [24], based on tests on copper-zinc alloy 69/31 CuZn specimens subjected to pre-strain carried out under constant-amplitude loading conditions (tension-compression) with controlled plastic strain amplitude ($\varepsilon_{ap}$ = const) and controlled stress amplitude ($\sigma_{a}$ = const), it was found that for small pre-strain (ε<20%) and small plastic strain levels ($\varepsilon_{ap}$ < 0.05%) a cyclic softening of the material occurs, while for high levels ($\varepsilon_{ap}$ > 0.1%)—unidirectional pre-strain led to its hardening.

Pre-strain may also change the course of cumulation of plastic strain $\varepsilon_{ap}$. This was observed, among other things, in [26] based on fatigue tests of pre-strained copper specimens under controlled plastic strain conditions. For low pre-strain levels, the material was characterized by cyclic hardening, while for high levels the material showed the characteristics of a cyclically softening material without a period of stabilization.

In [20] Brancoa et al. found that the initial deformation preceding the cyclic loading of the 7050-T6 aluminum alloy significantly changes the cyclic properties. In the absence of initial deformation, the aluminum alloy was characterized by cyclic softening. The application of the initial tensile stress gave a mixed effect, i.e., cyclic softening at higher strain amplitudes and cyclic hardening at lower strain amplitudes.

A separate research issue is the problem of the impact of initial deformations on the course of fatigue crack development. Based on the work by Schjjve [28], it can be concluded that initial strains cause a significant increase in the fracture rate. Similar conclusions were formulated in [20], where it was found that the level of initial deformation has a very significant influence on the rate of fracture. In the present paper, the authors considered the fatigue life up to fatigue crack initiation.

The aim of this paper is to evaluate the effect of pre-strain on the strength and low-cycle properties of specimens made of S420M grade steel. The scope of the tests was to determine the basic strength parameters of as-received (unstrained) and pre-strained specimens in static tensile and low-cycle fatigue tests. Conclusions were formulated on the basis of the analysis of hysteresis loop parameters recorded during fatigue tests and metallographic observations.

## 2. Materials and Methods

### 2.1. Description of Tests

#### 2.1.1. Test Specimens

**Figure 1:**
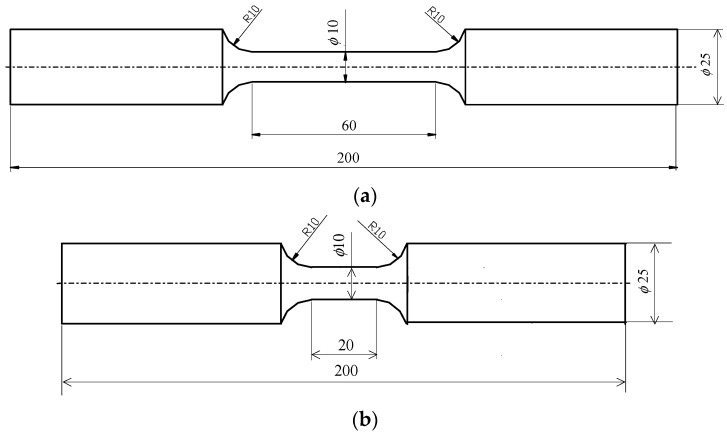
Test specimens: (**a**) for tensile testing, (**b**) for low-cycle fatigue testing (dimensions are given in mm).

The test specimens were made from a steel sheet of grade S420M (EN 10025-4) with a thickness of 30 mm. Table 1 summarizes the chemical composition of the steel. The test specimens (Figure 1) were prepared in accordance with standards [29,30,31].

Measurements of the diameters of the specimens were followed by measurements of the roughness $R_{a}$ of the measuring section, taken on a MarSurf XR 20 profilographometer. Measurements were taken on three randomly selected specimens, and an average parameter value of $R_{a}$ = 0.59 μm was obtained.

#### 2.1.2. Static Tests

##### (a) Tensile Tests

The tests consisted of subjecting five specimens to an incremental load at a piston travel speed of the testing machine of 0.05 mm/s. The tests were carried out on a hydraulic testing machine INSTRON 8502.

The elongation of the loaded specimen was measured using a static test extensometer (type 2630-110) with a base of 50 mm and a measuring range of +100%, attached to the measuring section of the specimen. The force was measured using a force gauge head (2518-113) with a measuring range of ±125 kN. Tensile tests were carried out at the temperature of 21 °C, and the instantaneous values of the loading force and elongation of the specimen were recorded during the tests. The tests were carried out until the specimen was permanently separated in the area of the measuring section.

##### (b) Tensile Tests with Unloading

Similar to the classical tensile tests with unloading, the tensile test was carried out on five specimens (Section 2.1.2), with the difference that, once elongation ε=10% was achieved, the specimen was unloaded and then loaded again until failure. Specimen strain and loading force were measured using the instrumentation used in the tests described in Section 2.1.2. During the tests, the instantaneous values of the loading force and elongation of the specimen were recorded.

#### 2.1.3. Low-Cycle Fatigue Tests©(c)

**Figure 2:**
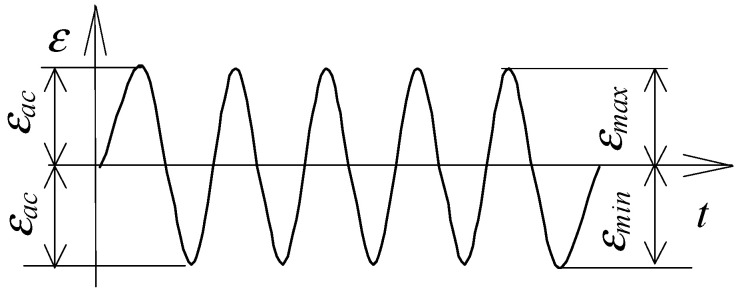
Load scheme.

The low-cycle fatigue tests used both specimens without straining (as-received specimens) and pre-strained specimens (ε=10%). The tests were carried out under controlled strain conditions ($\varepsilon_{ac}$ = const) for the cycle asymmetry factor $R=\varepsilon_{min}/\varepsilon_{max}=-1$, at five strain levels ($\varepsilon_{ac}$ = 0.25%, $\varepsilon_{ac}$ = 0.35%, $\varepsilon_{ac}$ = 0.5%, $\varepsilon_{ac}$ = 0.8%, $\varepsilon_{ac}$ = 1.0%). The strain levels ($\varepsilon_{ac}$) were adopted after analysing the static tensile graphs presented later in Section 3 of this paper). The load pattern is summarized in Figure 2.

The strain of the loaded specimen was measured using a dynamic test extensometer (type 2630-110) with a base of 10 mm and a measuring range of 1 mm, attached to the measuring section of the specimen. The force was measured using a force gauge head (2518-113) with a measuring range of ±125 kN. The occurrence of a visible fracture in the specimen was taken as the criterion for the end of the low-cycle fatigue tests. During the tests, the instantaneous values of the loading force and strain of the loaded specimens were recorded. The specimens were loaded at a frequency of f = 0.2 Hz. Fatigue tests were carried out in accordance with the standard [29]. At each deformation level, three fatigue tests were carried out. The points shown in the figures below correspond to the average values obtained at a given level of deformation.

## 3. Results

### 3.1. Static Tensile Tests

**Figure 3:**
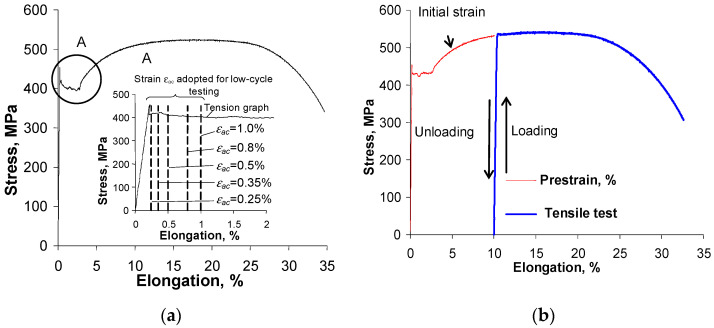
Tensile graphs: (**a**) classic tests, (**b**) tests with unloading.

The results of the classical tensile tests and the tensile tests with unloading are summarized in Figure 3 in the form of graphs in the coordinate system (total specimen strain ε, stress σ). For clarity, Figure 3 shows one example of each tensile graph. The stress in the specimen was calculated by dividing the value of the instantaneous load on the specimen by its initial cross-sectional area $S_{0}$. Figure 3a presents, in addition to the classical tensile graph, a section of the graph on which the strain levels adopted during the low-cycle fatigue tests are plotted. From an analysis of the graphs in Figure 3a, it can be concluded that the steel adopted for testing is characterized by the presence of a pronounced yield strength $R_{e}$.

At the stress level corresponding to yield strength $R_{e}$, there is a very pronounced increase in the elongation of the specimen (up to almost 3%) with no apparent change in load. Due to the amount of elongation of the specimen at this stage of the tensile test, all strain levels adopted for low-cycle fatigue testing ($\varepsilon_{ac}$) correspond to the yield strength. The mean values of the parameters determined in the classic tensile test and the tests with the unloading (from five repetitions) are summarized in Table 2.

The results of the tensile tests were subjected to statistical analysis. The analysis was based on a two-means test (for the group of results obtained in the classic tensile test and the results obtained in the tensile test with unloading). Due to the small number of specimens (n = 5 < 30), assumptions about the normality of the distributions of the test results were verified using the Shapiro-Wilk test, and assumptions about the equality of standard deviations of the results used the test for two variances. On the basis of the analyses carried out, it was concluded that, at the adopted level of significance (α = 0.05), the mean elongation $A_{5}$ of the specimens and the mean strength $R_{m}$ obtained in the classic test and the test with unloading can be considered statistically the same.

### 3.2. Low-Cycle Fatigue Tests

**Figure 4:**
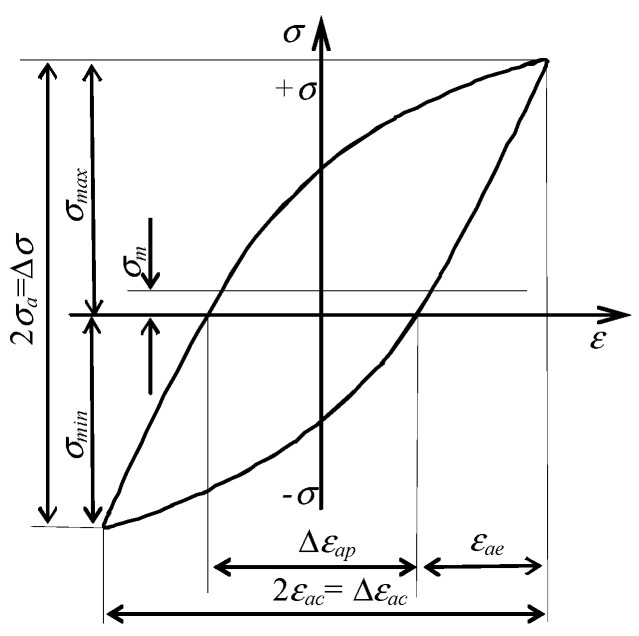
Hysteresis loop and its basic parameters.

The results of the low-cycle fatigue tests were analyzed in terms of changes in selected hysteresis loop parameters versus the number of a load cycle. The parameters adopted for the analysis are the stress amplitude $\sigma_{a}$ and the plastic strain range $\varepsilon_{ap}$. A graphical interpretation of the parameters adopted for the analysis is shown in Figure 4.

**Figure 5:**
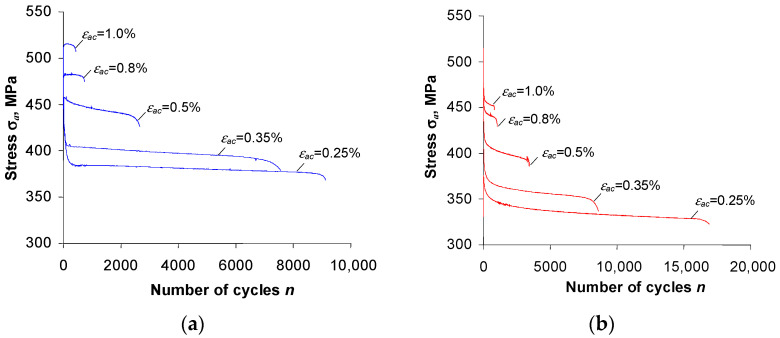
Stress amplitude $\sigma_{a}$ versus number of load cycle: (**a**) as-received specimens, (**b**) pre-strained specimens.

**Figure 6:**
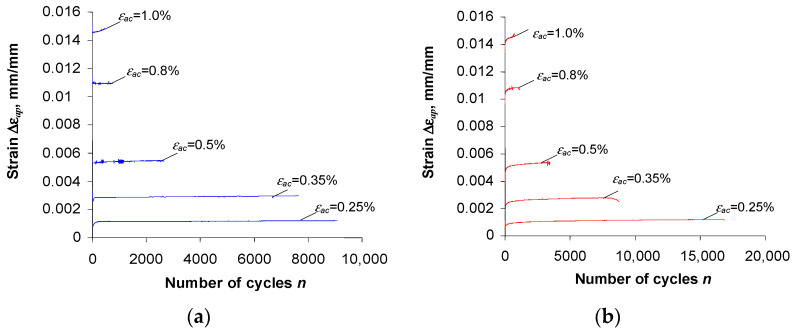
Plastic strain $∆\varepsilon_{ap}$ versus number of cycle: (**a**) as-received specimens, (**b**) pre-strained specimens.

Figure 5 and Figure 6 show the changes in stress $\sigma_{a}$ and plastic strain $∆\varepsilon_{ap}$ versus the number of a loading cycle, at five strain levels.

**Figure 7:**
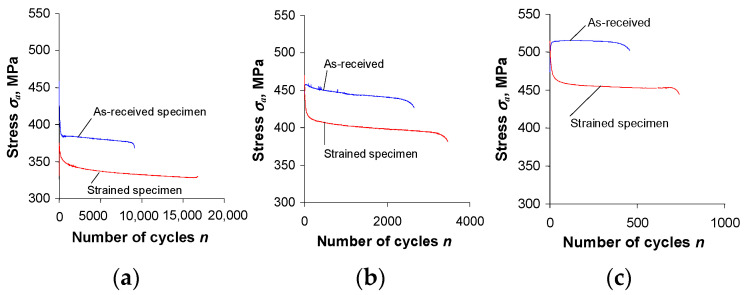
Influence of pre-strain on stress amplitude $\sigma_{a}$: (**a**) $\varepsilon_{ac}$ = 0.25%; (**b**) $\varepsilon_{ac}$ = 0.5%; (**c**) $\varepsilon_{ac}$ = 1.0%.

**Figure 8:**
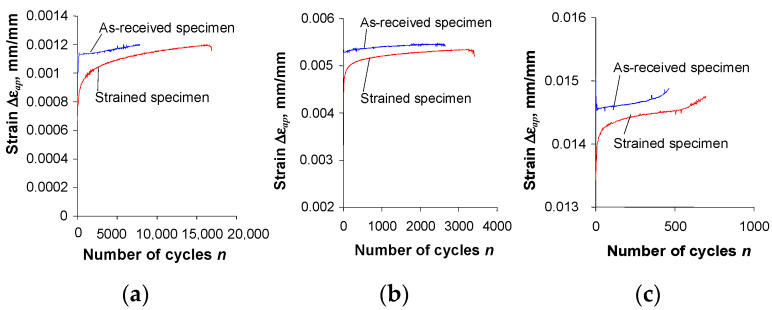
Influence of pre-strain on plastic strain $∆\varepsilon_{ap}$: (**a**) $\varepsilon_{ac}$ = 0.25%; (**b**) $\varepsilon_{ac}$ = 0.5%; (**c**) $\varepsilon_{ac}$ = 1.0%.

The analysis of the graphs (Figure 5 and Figure 6) shows that after the pre-straining of the specimens (ε = 10%), the stress amplitude $\sigma_{a}$ and plastic strain $∆\varepsilon_{ap}$ are—at identical levels of controlled strain, $\varepsilon_{ac}$—lower than the values of these parameters obtained for unstrained (as-received) specimens. To illustrate the magnitude of variation in the loop parameters, Figure 7 and Figure 8 present exemplary comparative graphs of $\sigma_{a}$ and $∆\varepsilon_{ap}$ for as-received and strained specimens at three strain levels $\varepsilon_{ac}$.

From the analysis of the graphs of the hysteresis loop basic parameters for the as-received specimens (Figure 5a), it can be concluded that the cyclic properties of the considered material depend on the strain level. In the strain range $\varepsilon_{ac}$ > 0.5%, the steel undergoes slight hardening. This is evidenced by a slight increase in stress at levels of $\varepsilon_{ac}$ = 0.8% and $\varepsilon_{ac}$ = 1.0%. At strain levels $\varepsilon_{ac}$ < 0.5%, the as-received specimens exhibit cyclic softening. This is confirmed by the reduction of stress amplitude $\sigma_{a}$ with the number of a cycle at these strain levels.

**Figure 9:**
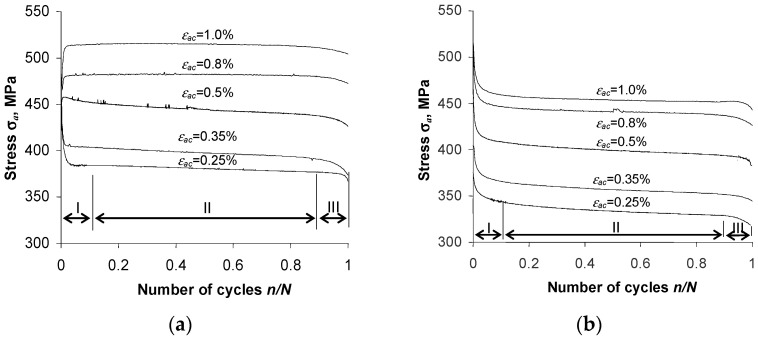
Stress variations $\sigma_{a}$ versus n/N at different strain levels $\varepsilon_{ac}$ : (**a**) as-received specimens, (**b**) strained specimens.

In the case of specimens undergoing pre-strain (Figure 5b), regardless of the level of $\varepsilon_{ac}$, S420M steel undergoes cyclic softening. This is evidenced by the decrease in stress $\sigma_{a}$ with the number of a load cycle, and a slight increase in plastic strain $∆\varepsilon_{ap}$. In order to compare the changes in cyclic properties of as-received and strained specimens at different strain levels $\varepsilon_{ac}$, Figure 9 presents graphs of stress $\sigma_{a}$ at five strain amplitude levels versus relative durability n/N.

In the stress waveforms $\sigma_{a}$ three characteristic stages can be distinguished:

Stage I—for pre-strained specimens (Figure 9b), the stage is characterized (independently of the strain level) by a rapid reduction in stress amplitude $\sigma_{a}$ (softening of the steel). For as-received specimens (Figure 9a), the course of change in cyclic properties at this stage is influenced by the level of strain $\varepsilon_{ac}$. For strain $\varepsilon_{ac}$ > 0.5% the stress $\sigma_{a}$ increases (cyclic hardening of the steel) while for strain $\varepsilon_{ac}$ < 0.5 the stress $\sigma_{a}$ decreases (cyclic softening of the steel). This stage covers a period of up to approximately 10% of the fatigue life and is associated with the degradation of material properties and the initiation of microcracks.

Stage II—for this stage and pre-strained specimens, a steady reduction in stress $\sigma_{a}$ is characteristic. For as-received specimens, as in Stage I, the course of change in cyclic properties is influenced by the level of strain $\varepsilon_{ac}$. For strain $\varepsilon_{ac}$ > 0.5% the stress $\sigma_{a}$ increases (hardening), while for strains $\varepsilon_{ac}$ < 0.5 the stress $\sigma_{a}$ decreases (softening of the steel). This stage is the longest and ranges from approximately 10% to 90% of fatigue life.

Stage III—in this stage, for both variants of steel specimens (as-received and pre-strained), a very rapid stress amplitude reduction is evident until the specimen fails.

A comparative analysis of the changes in the basic parameters of the hysteresis loop for as-received and pre-strained specimens (Figure 5 and Figure 7) also highlighted the effect of pre-straining on the value of the mean stress $\sigma_{m}$. A graphical interpretation of stress $\sigma_{m}$ during low-cycle tests at $\varepsilon_{ac}$ = const is shown in Figure 5, and is calculated as:

(1) $$\sigma_{m}=\frac{\sigma_{max}+\sigma_{min}}{2}$$

**Figure 10:**
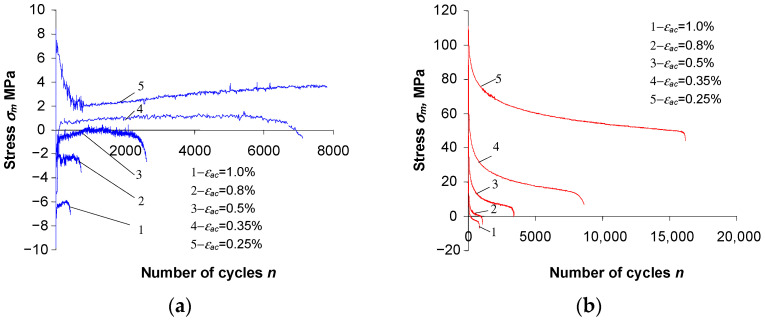
Changes in mean stress $\sigma_{m}$ versus number of cycle: (**a**) as-received specimens, (**b**) pre-strained specimens.

During low-cycle testing of as-received specimens, the mean stress $\sigma_{m}$ was low (Figure 10a). Its value at certain levels of strain amplitude is close to the error of measurement of the force loading the specimen. Despite the small values, the stress $\sigma_{m}$ during testing of as-received specimens decreases with increasing strain $\varepsilon_{ac}$. On the other hand, for pre-strained specimens the mean stress $\sigma_{m}$ is significantly higher. It reaches its highest values at the lowest strain levels ($\varepsilon_{ac}$ = 0.25%) and, as with strain $\sigma_{a}$, it decreases with the number of a load cycle. Figure 10 shows the changes in mean stress $\sigma_{m}$ during testing of as-received and pre-strained specimens.

The pronounced stress asymmetry during cyclic loading of pre-strained specimens can be attributed to the occurrence of the Bauschinger effect [32]. The pre-strain with a simultaneous exceeding of the yield strength in tension ($\varepsilon_{ac}$ = 10%) resulted in a decrease in the yield strength in compression conditions and thus an increase in the mean stress $\sigma_{m}$.

**Figure 11:**
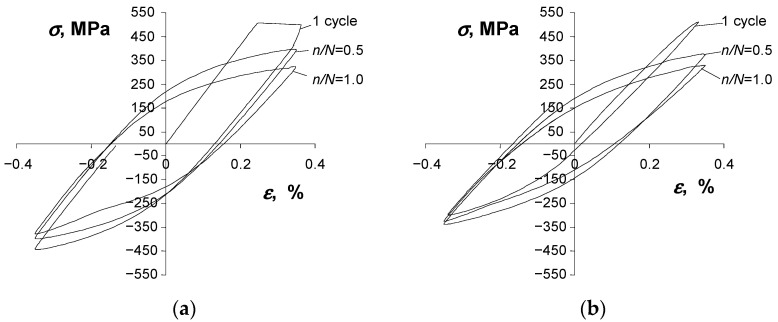
Hysteresis loops at $\varepsilon_{ac}$ = 0.35%: (**a**) as-received specimen, (**b**) pre-strained specimen.

**Figure 12:**
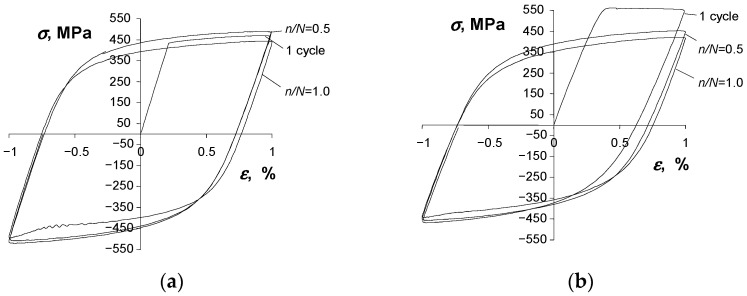
Hysteresis loops at $\varepsilon_{ac}$ = 1.0%: (**a**) as-received specimen, (**b**) pre-strained specimen.

A consequence of changes in the basic loop parameters versus the number of a load cycle is also the change in the shape of a hysteresis loop. Figure 11 and Figure 12 show the hysteresis loops recorded at two strain levels ($\varepsilon_{ac}$ = 0.25% and $\varepsilon_{ac}$ = 1.0%) at three different life stages, i.e., at the beginning of the test (n = 1), at mid-life (n/N = 0.5), and at the end of the fatigue test (n/N = 1).

A comparative analysis of the loops shown in Figure 11 and Figure 12 confirms that the properties of the as-received, as well as the pre-strained specimens, change during testing. Regardless of the condition of the specimen, there is no clear period of stabilization, which significantly hinders the development of test results.

**Figure 13:**
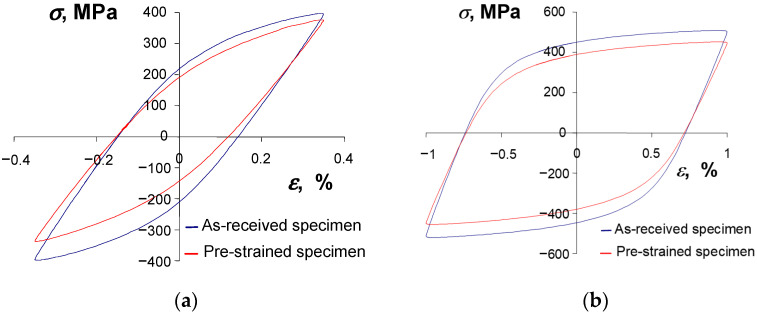
Hysteresis loops from period n/N = 0.5: (**a**) $\varepsilon_{ac}$ = 0.35%, (**b**) $\varepsilon_{ac}$ = 1.0%.

According to standard [29], in the absence of a clear stabilization period, the low-cycle properties are determined from a period corresponding to half-life (n/N = 0.5). To compare the cyclic properties of the strained and as-received specimens, Figure 13 shows the hysteresis loops from a period corresponding to half-life (n/N = 0.5).

The shape and parameters of the hysteresis loop (Figure 13) confirm the test results shown in Figure 7 and Figure 8. After pre-strain, the stress $\sigma_{a}$ decreases and the plastic strain range $∆\varepsilon_{ap}$ also decreases slightly. To describe the analytical relationship between stress $\sigma_{a}$ and strain $\varepsilon_{ap}$, an equation of the form proposed in [29] is adopted:

(2) $$lg\sigma_{a}=lgK^{\prime }+n^{\prime }lg\varepsilon_{ap}$$

**Figure 14:**
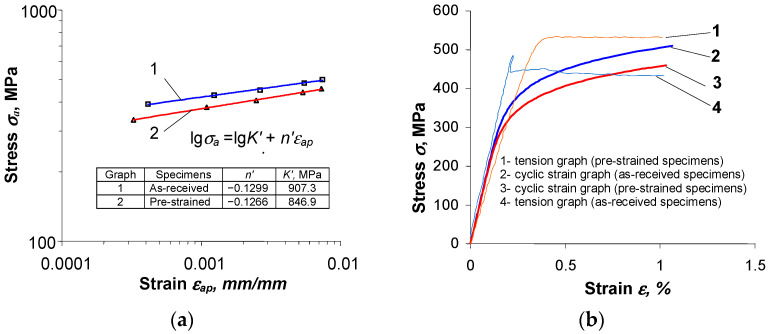
Results of tests on raw and pre-strained specimens: (**a**) relationship between stress $\sigma_{a}$ and strain $\varepsilon_{ap}$, (**b**) tensile and cyclic strain graphs.

Values of the loop parameters $\sigma_{a}$ and $\varepsilon_{ap}$ were developed using the least squares method by determining the coefficients and exponents of the simple regression described by Equation (2). The strain graph, obtained by approximating the loop parameters ($\sigma_{a}$ and $\varepsilon_{ap}$) from periods corresponding to half the fatigue life, is shown in Figure 14. Equation (2) and its parameters are also included in the figure. Figure 14b shows the cyclic strain graphs approximated by the equation proposed by Ramberg-Osgood [33] as follows:

(3) $$\varepsilon_{ac}=\frac{\sigma_{a}}{E}+{\left(\frac{\sigma_{a}}{K^{\prime }}\right)}^{\frac{1}{n^{\prime }}}$$

The influence of strain level $\varepsilon_{ac}$ on the cyclic properties of the as-received specimens is confirmed by the mutual position of the cyclic strain and tensile graphs. For as-received specimens, in the strain range $\varepsilon_{ac}$ < 0.5% the cyclic strain graph 2 in Figure 14b is located below the static tensile graph 4, while for strains of $\varepsilon_{ac}$ > 0.5% the cyclic graph is located above the static graph. For pre-strained specimens, the cyclic strain graph described by Equation (3) lies below the static tensile graph, confirming the softening of this material over the entire range of strain amplitudes.

On the basis of the tests carried out, it was found that at the same strain levels $\varepsilon_{ac}$, the durability of the pre-strained specimens was always higher than that of the as-received specimens. The increase in durability is a consequence of the reduction in stress $\sigma_{a}$ and strain $\varepsilon_{ap}$ due to the pre-strain of the specimens. The above confirms the test results observed during the tests of the TRIP multi-phase steel described in [22].

**Figure 15:**
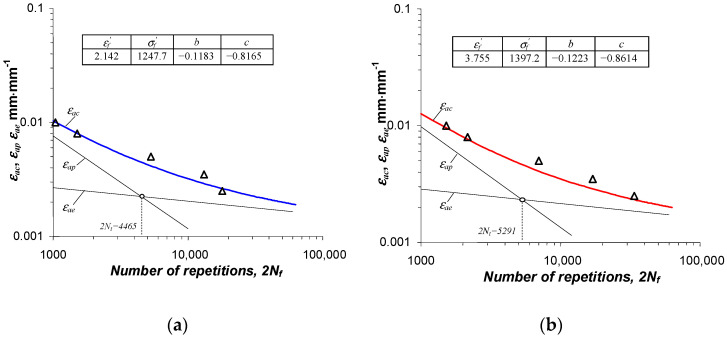
Fatigue graphs: (**a**) as-received specimens, (**b**) pre-strained specimens.

The increase in durability of pre-strained specimens is more pronounced at lower strain levels $\varepsilon_{ac}$. As the strain increases, the variation in the obtained durabilities decreases. The fatigue life results obtained in this study are summarized as graphs in the $2N_{f}-\varepsilon$ coordinate system in Figure 15. Bilogarithmic fatigue graphs were approximated by an equation in the form of [31]:

(4) $$\frac{∆\varepsilon_{ac}}{2}=\frac{∆\varepsilon_{ae}}{2}+\frac{∆\varepsilon_{ap}}{2}=\frac{{\sigma^{\prime }}_{f}}{E}{\left(2N_{f}\right)}^{b}+{\varepsilon^{\prime }}_{f}{\left(2N_{f}\right)}^{c}$$

**Figure 16:**
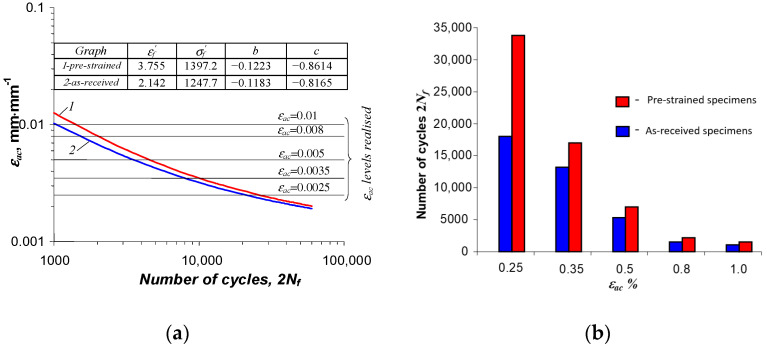
Results of low-cycle fatigue testing of as-received and pre-strained specimens: (**a**) fatigue graphs, (**b**) durability comparison.

The values of the exponents and coefficients of Equation (4) are tabulated in Figure 15. For comparison, Figure 16 summarizes the test results obtained with as-received and pre-strained specimens in a single coordinate system.

Based on a comparative analysis of the durabilities obtained using as-received and pre-strained specimens, it can be concluded that preceding the fatigue test with a permanent strain increases the fatigue life of the specimen relative to that of the as-received specimen. The largest increase in life (almost 100%) was observed at the lowest strain level ($\varepsilon_{ac}$ = 0.25%). A much smaller (around 20%) increase in life was observed at $\varepsilon_{ac}$ = 1.0%.

It should be remembered that the tests presented in the paper were carried out under the conditions of controlled deformation ($\varepsilon_{ac}$ = const). Such test conditions make it possible to avoid the so-called cyclic creep, which accompanies low-cycle tests conducted under conditions ($\sigma_{a}$ = const). In operating conditions, objects are most often subject to loads, where the controlling variable is force or stress. In works [34,35,36,37], it was found that e.g., in the conditions of controlled stress ($\sigma_{a}$ = const) the durability is usually lower than the results obtained in the conditions of controlled deformation ($\varepsilon_{ac}$ = const). For this reason, it is not possible to generalize the conclusions to the entire area of low-cycle fatigue. To generalize the conclusions, it is necessary to carry out studies on the impact of initial deformations under controlled stress conditions.

## 4. Microstructural Observations

**Figure 17:**
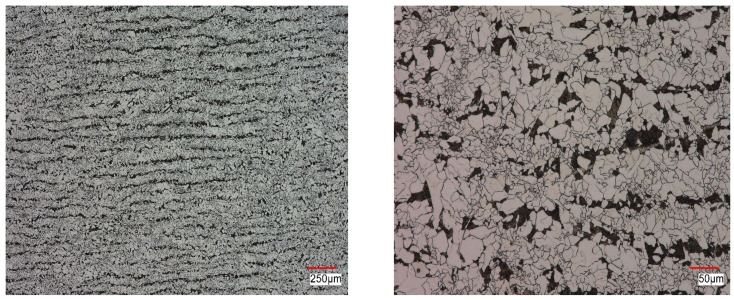
S420M steel structure in as-received state presented in two magnifications.

The S420M steel in the as-received state was characterized by a ferritic-pearlitic microstructure with a varied grain size in the cross-section (Figure 17). Grain size estimated using drawing standards was 9-7 for ferrite, 6 for single grains, and 8/7 for pearlite. In addition to polygonal ferrite grains, some of them were similar in character to bainitic ferrite, and single ferrite grains of plate and strip shape were also observed. A large dispersion of cementite was observed in the pearlite. Pearlite was isolated in the form of separate colonies, and in some places, pearlite grains were observed separated along ferrite grain boundaries. The above structure indicates an accelerated cooling of the tested material after the plastic working process; moreover, it may indicate an anisotropy of properties.

**Figure 18:**
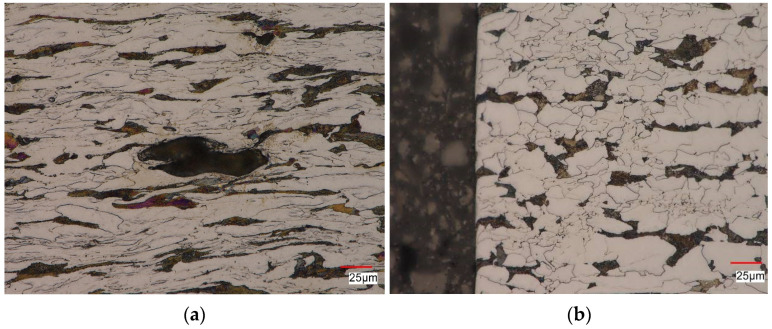
Structure of S420M steel: (**a**) after static tensile test, (**b**) after initial deformation equal to 10%.

As expected, after the static tensile test, a very strongly deformed ferritic-pearlitic band microstructure with a characteristic texture was observed (Figure 18). Steel flow took place in the direction of the principal stress. Numerous microvoids were observed at the pearlite/ferrite interface or in the areas of pearlitic colonies. In the pre-deformed material, a microstructure similar to the material in the as-received condition was also observed. In contrast to the material after the tensile test (Figure 18a), after initial deformation (Figure 18b), only single ferrite grains elongated in the direction of material flow were observed.

**Figure 19:**
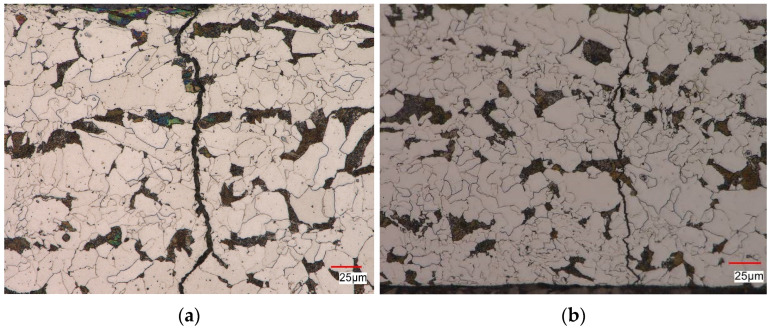
Structure of S420M steel after fatigue test at $\varepsilon_{ac}$ = 1.0%: (**a**) non-pre-deformed sample, (**b**) pre-deformed sample (10%).

Figure 19 shows an example of the structure obtained during fatigue loads at the deformation level $\varepsilon_{ac}$ = 1.0%, using a non-pre-deformed sample (Figure 19a) and a pre-deformed sample (Figure 19b). In the case of non-pre-deformed samples, a ferritic-pearlitic microstructure with characteristics similar to the material in the as-received state was observed near the crack (Figure 19a). The cracks in the sample propagated along the ferrite and pearlite grain boundaries and developed perpendicularly to the axis of the sample. Cracks propagating parallel to the scrap surface were also visible.

In the case of the pre-deformed sample (Figure 19b), the microstructure near the surface of the scrap was similar to the material in the as-received state. The revealed cracks nucleated on the side surface of the sample and propagated both along the grain boundaries and through the ferrite and pearlite grains. As in the case of a non-pre-deformed material, cracks propagating parallel to the scrap surface were initially observed.

**Figure 20:**
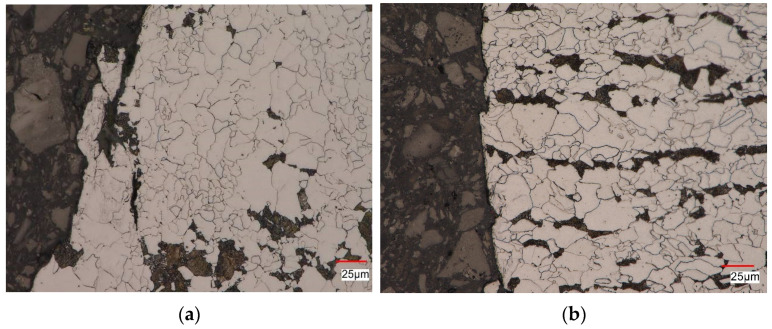
Structure of S420M steel after fatigue test at $\varepsilon_{ac}$ = 0.35%: (**a**) non-pre-deformed sample, (**b**) pre-deformed sample (10%).

Figure 20 shows an example of the microstructure of the tested steel obtained under fatigue loads at the deformation level $\varepsilon_{ac}$ = 0.35% using a non-pre-deformed sample (Figure 20a) and a pre-deformed sample (Figure 20b). In both analyzed materials, a ferritic-pearlitic microstructure with characteristics similar to the material in the as-received state was observed near the surface of the scrap. At the surface of the scrap, cracks propagating parallel to this surface were observed.

## 5. Conclusions

The static tensile and low-cycle fatigue tests carried out on S420M steel specimens allow several conclusions to be drawn, the most important of which are:

(a) A pre-strain of S420M steel specimens of almost 33% of the total elongation ($A_{5}$) does not reduce the basic strength parameters determined in the static tensile test.
(b) The cyclic properties of as-received S420M steel specimens depend on the level of strain. For strains $\varepsilon_{ac}$ > 0.8% the steel is subject to cyclic hardening, while for strains of $\varepsilon_{ac}$ < 0.8% the steel is characterized by cyclic softening. Pre-strained S420M steel specimens, independently of the level of alternating load, are subject to cyclic softening.
(c) Preceding the low-cycle test with a pre-strain of the specimens (ε = 10%) results in stress asymmetry. The magnitude of the mean stress $\sigma_{m}$ is affected by the level of strain $\varepsilon_{ac}$. Mean stress $\sigma_{m}$ reaches its highest values at the lowest strain level ($\varepsilon_{ac}$ = 0.25%).
(d) A comparative analysis of the basic parameters of the hysteresis loop for as-received and pre-strained specimens made of S420M steel, at the same strain levels of $\varepsilon_{ac}$, showed that pre-straining results in a reduction of two basic hysteresis loop parameters, i.e., the stress $\sigma_{a}$ and the plastic strain range $∆\varepsilon_{ap}$.
(e) The consequence of decreasing the stress amplitude $\sigma_{a}$ and plastic strain range $∆\varepsilon_{ap}$ is an increase in the fatigue life of pre-strained specimens relative to that of as-received specimens. The increase in durability of pre-strained specimens is influenced by the level of total strain. The greatest increase was observed at the strain level $\varepsilon_{ac}$ = 0.25%.
(f) The structure of S420M steel in the as-received state indicates an accelerated cooling of the tested material after the plastic working process. This could be the reason for the anisotropy of the steel properties, which caused a slight dispersion of the results of fatigue tests of undeformed samples as well as pre-deformed samples.
(g) The cracks of the S420M steel samples in the non-pre-deformed and pre-deformed state nucleated on the surface of the sample and propagated both along the grain boundaries and through the ferrite and pearlite grains. For both conditions of the samples, cracks propagating parallel to the surface of the fatigue scrap were also observed.

The analysis of the test results proved that the occurrence of an overload during operation does not necessarily rule out the continued operation of the technical facility. The static properties, as well as the fatigue properties of S420M steel after overloading, are not lower than those specified for the as-received material.

## Figures and Tables

**Table 1:** Chemical composition of S420M steel in weight percent.

Fe	C	Si	Mn	P	Cr	Al	Nb	Ti	V	W
98.0	0.125	0.215	1.45	0.0135	0.0208	0.0268	0.0288	0.013	0.0519	0.0150

**Table 2:** Static test results.

Test Type	$A_{5},\;\%$	$R_{m},\;\mathrm{MPa}$	$R_{eH},\;\mathrm{MPa}$	$R_{eL},\;\mathrm{MPa}$	E, Mpa
Classic test	32.2	527.3	434.5	398.3	2.1 × 10^5^
Test with unloading	33.3	528.7	-	-	-

## Data Availability

Data sharing not applicable.
